# Dihydroartemisinin Potentiates VEGFR-TKIs Antitumorigenic Effect on Osteosarcoma by Regulating Loxl2/VEGFA Expression and Lipid Metabolism Pathway

**DOI:** 10.7150/jca.81623

**Published:** 2023-03-27

**Authors:** Xiaomin Ding, YaWen Zhang, Jinrong Liang, Qian Li, Haiyan Hu, Yan Zhou, Bing Zhang

**Affiliations:** 1 Orthopaedic Department of the Affiliated Hospital of Jiangxi University of Traditional Chinese Medicine, Nanchang, 330006, China; 2 Department of Oncology, Shanghai Jiao Tong University Affiliated Sixth People's Hospital, Shanghai, 200233, China.

**Keywords:** Dihydroartemisinin, antiangiogenic drug, synergy, drug-resistance, LOXL2

## Abstract

Anti-angiogenesis therapy has shown significant anti-tumor effects against a variety of cancers. But resistance to antiangiogenic drugs, intrinsic and evasive, is frequently found in patients during treatment. Here, we report that dihydroartemisinin (DHA), a derivative of the Chinese medicine artemisinin, enhances antiangiogenic drug-induced cytotoxicity in osteosarcoma (OS) cells. Proteomics analysis revealed that DHA treatment significantly affected the activity of the collagen-modifying enzyme lysyl oxidase-like 2 (LOXL2), a regulatory gene associated with poor prognosis of OS. Furthermore, we found that DHA reduced the expression of vascular endothelial growth factor (VEGFA) by downregulating LOXL2. This mechanism was confirmed by QRT-PCR, western blot, and ELISA assays. Correspondingly, vector-enforced expression of LOXL2 markedly reduced VEGFA secretion. Untargeted metabolomic analysis revealed that the lipid metabolism that confers antiangiogenic drug resistance, was also interfered with by DHA. Thus, DHA not only exerts antitumor effects in OS cells directly but also synergizes with the antiangiogenic drug by regulating vascular endothelial growth factor A (VEGFA) expression and lipid metabolism.

## Introduction

Osteosarcoma (OS) is the most common primary bone tumor. It tends to occur during childhood and adolescence and generally arises in the extremities 1. Currently, anthracycline-based chemotherapy and surgery are the standard treatment for OS. For localized osteosarcoma, the 5-year survival rate can be enhanced from generally around 20% to 60-70% by surgical resection plus sequential systemic multi-drug chemotherapies. However, patients with recurrence or metastasis still have the lower overall 5-year survival rates of about 20% 2, underlining the need for an effective new anticancer option for OS.

Dihydroartemisinin (DHA), one of the artemisinin derivatives, has been used as first-line anti-malarial therapy due to its lower toxicity, higher oral bioavailability, and better antimalarial effect than artemisinin. In addition to antimalarial activity, DHA has also been shown to exert beneficial effects on inflammatory diseases such as inflammatory bowel disease, osteoarthritic synovium, rheumatoid arthritis, and systemic lupus erythematosus 3,4,5. Specifically, several reports showed that DHA could effectively inhibit the proliferation of tumor cells, including those of leukemia, ovarian cancer, breast cancer, liver cancer, and other solid tumors. Gao et al. reported that DHA induced apoptosis of the leukemia cells through Mcl-1 down-regulation, and MEK/ERK inactivation 6. DHA induction of autophagy in liver cancer cells via the AKT-mTOR pathway has also been reported 7. These earlier studies of DHA antitumor function indicated multiple signaling pathways, mostly inducing cell cycle arrest, apoptosis, and ferroptosis, thus these appear to be the most common mechanisms in most tumors. Studies of DHA in osteosarcoma are rare but Shen et al. found that the amount of iron in osteosarcoma cells was higher than that in normal bone cells and iron could promote dihydroartemisinin cytotoxicity via ROS production in a dose-dependent manner. The authors also observed that DHA could damage lysosomes to result in autophagic flux blockage 8. Thus, multiple reports have indicated that DHA acts through several pathways to prevent the development of a range of tumors.

For osteosarcoma, there is no doubt that tumor neovascularization induces tumor growth and metastasis. The blockage of neovascularization by drugs with specific targets is termed antiangiogenic treatment. Antiangiogenic drugs target the vascular endothelial growth factor (VEGF) and/or VEGF receptor. Recently, vascular endothelial growth factor receptor-tyrosine kinase inhibitors (VEGFR-TKIs) have been brought into use, individually or in combination therapy, as second-line or subsequent treatments to compensate for resistance to chemotherapy and improve outcomes in OS patients. However, the receptors targeted by these drugs can control not only angiogenesis but also other physiological processes such as coagulation and blood pressure 9, consequently, there is a tendency to reduce the oral dosage or even suspend the treatment because of intolerable adverse events during the treatment. Besides frequent adverse events, there is often a low therapeutic efficacy as patients always develop intrinsic resistance to antiangiogenic drugs.

In this work, we treated OS cells in vitro with combined DHA and VEGFR-TKIs and uncovered a surprisingly synergistic antitumor effect. We describe the mechanisms underlying the beneficial effect of this combination procedure: DHA inhibits the VEGFA expression by downregulating LOXL2 to reduce angiogenesis. Furthermore, DHA could reverse antiangiogenic drug resistance by inhibiting fatty acid oxidation, which is an essential component of lipid metabolism reprogramming. Our study demonstrated the preclinical potential of combining DHA and VEGFR-TKIs in OS and this was verified by in vivo experiments.

## Materials and methods

### Cell culture and cell viability assay

Human Saos-2 and 143b OS cells were obtained from the Chinese Academy of Sciences. Cells were cultured in DMEM containing 10% FBS (Gibco, CA, USA) in a humidified atmosphere containing 5% CO^2^ at 37℃. DHA, apatinib, anlotinib, and sorafenib were purchased from Selleck Chemicals (Houston, TX, USA). These agents were dissolved in DMSO (Sigma-Aldrich, St. Louis, MO, USA). OS cells were seeded into 96-well plates. After treatment, CCK-8 was added and incubated with cells according to the instructions of the CCK-8 kit (CCK8; Dojindo, Kumamoto, Japan). The absorbance was measured at 450 nm.

### Drug combination analysis

The synergy between DHA and the VEGFR-TKIs was assessed using the methodology proposed by Chou and Talalay 10. Drug concentrations were in a series of 2-fold dilutions above and below the IC_50_ of each drug. One day after seeding, cells were treated with DHA, apatinib, anlotinib, and sorafenib alone or in combination for 24 h. The combination index was calculated by CalcuSyn, v2.0 (Biosoft). CI < 0.9 indicates synergism, CI = 0.9-1.1 indicates nearly additive, CI > 1.1 indicates antagonism.

### Apoptosis and cell cycle assay by FCM

Saos-2 and 143b cells were seeded in 6-cm plates and incubated with DHA and apatinib. After 24 h, cells were harvested and stained with Annexin V-FITC/PI apoptosis detection kit (BD Biosciences Pharmingen, San Diego, CA, USA). All resulting fluorescence was assessed by fluorescence-activated cell sorting scan (FACS) flow cytometry (Becton Dickinson, Mountain View, CA, USA).

### Enzyme‑linked immunosorbent assay (ELISA)

The levels of VEGFA and CPT-1 were detected using an ELISA kit (Wuhan Guge Biological Technology Co., Ltd.) according to the manufacturer's protocols. Standard curves were created using purified VEGFA or CPT-1.

### Wound healing assay

Cells were seeded into 6-well plates and cultured overnight. The wounds were scratched with a 200-µl pipette tip. Then, the cells were washed with PBS to remove the suspended cells and cultured continuously. 24 h later, the wounds were photographed under a microscope (Olympus, Tokyo, Japan).

### Transwell assay

After being treated with DHA and apatinib for 24 h, 1×10^5^ viable Saos-2 and 143b cells were seeded in Matrigel-coated Boyden chambers containing serum-free DMEM medium in the upper chamber and complete medium in the lower compartment for 24 h. Then the migrated cells were fixed with 4% paraformaldehyde for 15 min and stained with 0.1% crystal violet for 20 min. The invading cells were counted in five random fields under a microscope (Olympus, Tokyo, Japan).

### Label-free quantitative proteomics

To explore the underlying networks regulated by DHA, label-free based quantitative proteomic analysis was carried out on Saos-2 cells treated with IC50 (25 μM) of DHA for 24 h. SDT buffer (4% SDS, 100 mM DTT, 150 mM Tris-HCl pH 8.0) buffer was used for cell lysis and protein extraction. Quantitative proteomic analysis was performed at Shanghai Applied Protein Technology Co., Ltd. The peptides were separated by an Ultra-High Performance Liquid system (Thermo Scientific, USA), and then subjected to LC-MS/MS analysis on a Q Exactive HF-X mass spectrometer (Thermo Scientific) for 60 min. The MS raw data for each sample were combined and searched using the MaxQuant 1.5.3.17 software for identification and quantitation analysis. All identified proteins were determined using a false discovery rate (FDR) threshold of ≤ 0.01.

### Untargeted metabolomic relative quantitative analysis

Untargeted metabolomics profiling of Saos-2 cells treated with DHA or DMSO was performed using a quadrupole time-of-flight system (AB Sciex TripleTOF 6600, AB SCIEX) coupled with ultra-high-performance liquid chromatography (1290 Infinity LC, Agilent Technologies) at Shanghai Applied Protein Technology Co., Ltd. The samples were mixed with 400 μL of cold methanol/acetonitrile (1:1, v/v) to remove the protein and centrifuged for 20 minutes. After sample normalization, the data were exported to the SIMCA-P software for multivariate data analysis to screen for differential metabolites.

### qRT-PCR assay

The mRNA levels of GAPDH, LOXL2, VEGFA, CPT1A, ACSL1, and ACAT1 were detected with a PrimeScript RNA RT-PCR Kit (Sangon Biotech, Shanghai, China), following the manufacturer's protocol. The primers are shown in S1. The relative mRNA levels were calculated using the 2^-ΔΔCt^ method.

### Western blot assay

The expression of proteins was quantified with a BCA Protein Assay Reagent Kit (Thermo Fisher, USA). The rabbit polyclonal secondary antibody was purchased from Cell Signaling Technology (Boston, USA). GAPDH, LOXL2, VEGFA, CPT1A, ACSL1, and ACAT1 antibodies were purchased from Proteintech Group, Inc. (Chicago, IL, USA). The band density was quantified using ImageJ software.

### Construction of the plasmids and transfection

The loxl2 overexpression pcDNA3.1 plasmid was constructed by RiboBio Co., Ltd. (Shanghai, China). The plasmid was transfected into Saos-2 and 143b cells using Lipofectamine 3000 (Invitrogen) following the manufacturer's protocol.

### Mice xenografts

The animal study was performed in accordance with the NIH Guide for the Care and Use of Laboratory Animals approved by the Scientific Investigation Board of Shanghai Jiao Tong University Affiliated Sixth People's Hospital. Five-week-old female BALB/c nude mice with a weight range, of 26-31g were prepared. 143b cell-derived xenografts were allowed to grow to an average volume of 200 mm^3^ and then treated intraperitoneally with DMSO, apatinib or DHA alone (50 mg/kg), and DHA (25 mg/kg) combined with apatinib (25 mg/kg) every 3 days. After 30 days of treatment, the tumors were measured and calculated according to this formula: volume = length * width^2^/2.

### Bioinformatics analysis and statistics

For proteomics analysis, differentially expressed proteins (DEPs) were filtered by *p* < 0.05 and the fold change value > 2.0 or < 0.5. Metascape database was used as gene sets for GO and KEGG pathway enrichment analysis. Significantly enriched pathways were chosen at a level of 0.05 normal *p*-value and 0.25 FDR q value. For metabolomics analysis, differentially expressed metabolites were calculated by unpaired Student's t-test. The variable importance in the projection (VIP) value > 1 and *p* < 0.05 was significant. The KEGG database was selected to note the metabolites and enrichment analysis. Only pathways with *p* < 0.05 were considered as significantly changed pathways. The non-omics data were analyzed by Student's *t*-test using GraphPad Prism 5.0. *p* < 0.05 was considered statistically significant and continuous variables are presented as the mean ± SD.

## Results

### DHA inhibits proliferation, migration, invasion, and induces apoptosis in OS cells

**Figure 1:**
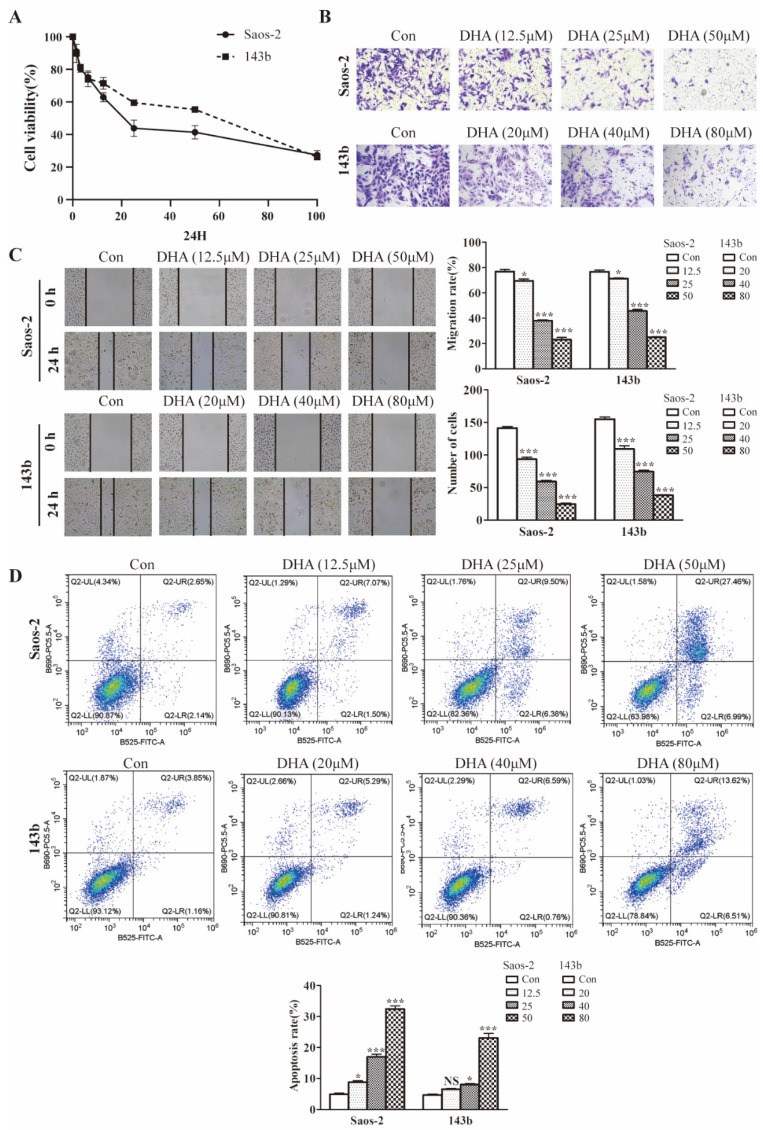
DHA inhibits proliferation, migration, invasion, and induces apoptosis in OS cells. (A) After exposure to various concentrations (1.5-100 μM) of DHA for 24 h, the viability of Saos-2 and 143b cells was assessed by a CCK-8 assay. The cell viability decreased dose- and time-dependently. (B) After incubation with various concentrations of DHA (12.5, 25, 50 μM for Saos-2 and 20, 40, 80 μM for 143b), the transwell assays indicated that the invasion rates of OS cells decreased dose-dependently in the DHA groups. (C) The wound healing assay showed that DHA impaired wound healing dose-dependently. (D) OS cells were stained with Annexin V-FITC and PI and then subjected to flow cytometry analysis of cell apoptosis, DHA induced OS cells apoptosis dose-dependently. (* *P* < 0.05, *** *P* < 0.001 compared with the control).

The effect of DHA on cell proliferation was tested using cell testing kit CCK8. As shown in the picture (Figure 1A), 143b and Saos-2 cells were significantly inhibited in a dose-dependent manner. The IC_50_ at 24 h of treatment was 41.98 µM for 143b and 25.41 µM for Saos-2 cells. Wound healing and transwell assays were performed to test for effect on the migratory and invasive abilities. Compared with the control group, the invasion rates of the DHA treatment group were decreased (Figure 1B). The wound healing assay indicated that migration rates in the DHA treatment group were much less than those in the control group (Figure 1C). Flow cytometry showed the function of DHA in promoting OS cell apoptosis (Figure 1D).

### LOXL2 is a potential drug target of DHA that is essential for OS cells growth

**Figure 2:**
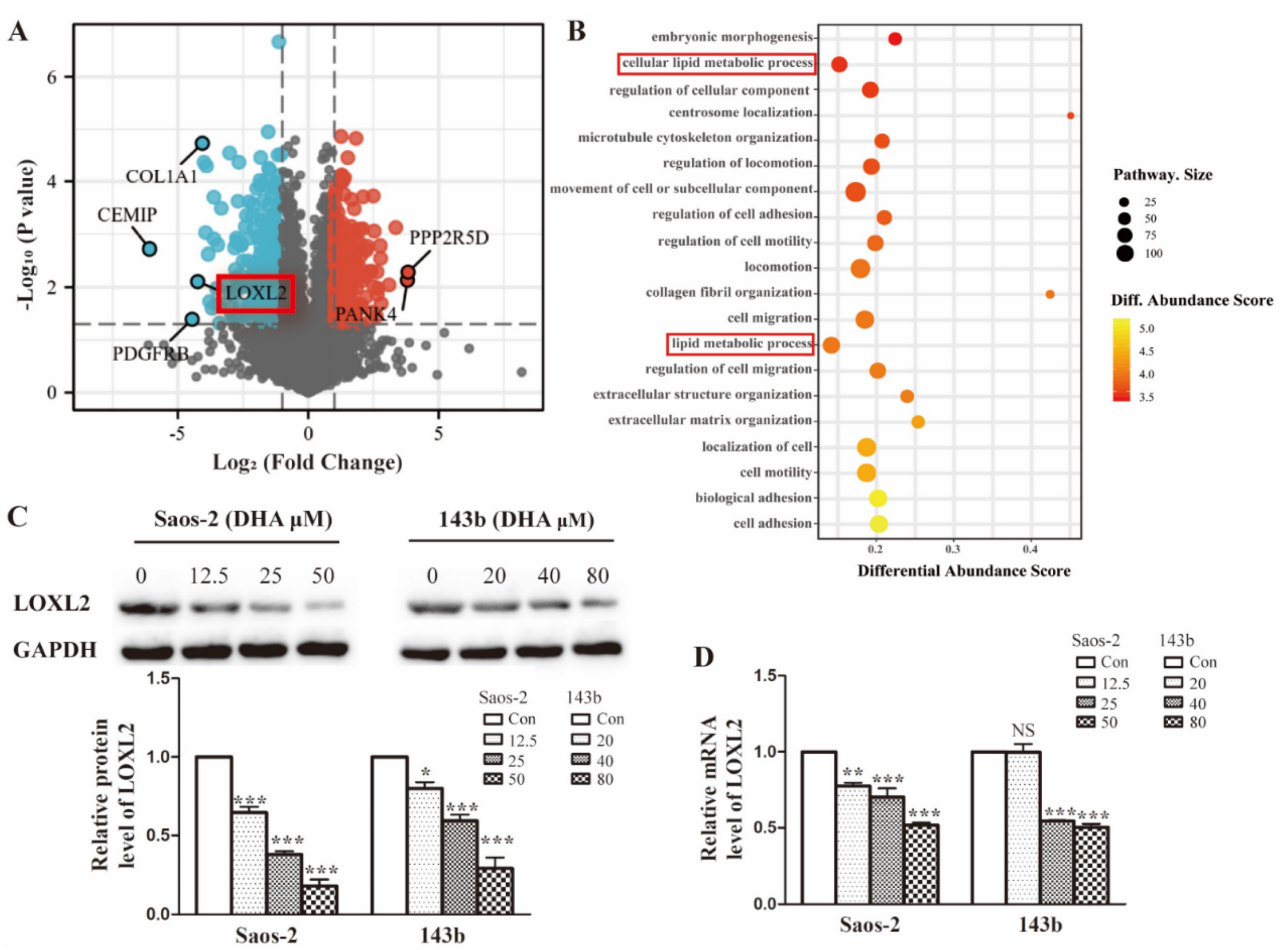
LOXL2 is a potential drug target of DHA essential for OS cells growth. (A) Volcano plot of the global profile of proteins after DHA treatment, proteins marked in black are the most influenced. (B) Gene ontology (GO) annotation shows 20 significantly enriched pathways after DHA treatment. (C) The protein levels of LOXL2 were downregulated by DHA dose-dependently in Saos-2 and 143b cells. (D) qRT-PCR showed that the LOXL2 mRNA levels were downregulated by DHA dose-dependently in Saos-2 and 143b cells (^NS^ *P*>0.05, * *P* < 0.05, ** P < 0.01, *** P < 0.001 compared with the control).

To investigate the possible molecular basis of DHA targeting in osteosarcoma, we performed proteomic analysis of Saos-2 cells treated with DHA for 24 h. In total, 5053 proteins were quantified and 594 differentially expressed proteins (DEPs) were filtered by the criteria *p* < 0.05 and fold change value > 2.0 or < 0.5. A volcano plot shows the top up-regulated and down-regulated proteins (Figure 2A). The classification of DEPs by GO database annotation showed that the lipid metabolic processes were significantly enriched (Figure 2B). To further evaluate the change in LOXL2 expression induced by DHA, qRT-PCR and western blot assays were performed. In line with the proteomic results, LOXL2 was confirmed to be downregulated by DHA dose-dependently (Figure 2C and Figure 2D).

### Treatment with DHA leads to VEGFA reduction via LOXL2/VEGFA pathway

**Figure 3:**
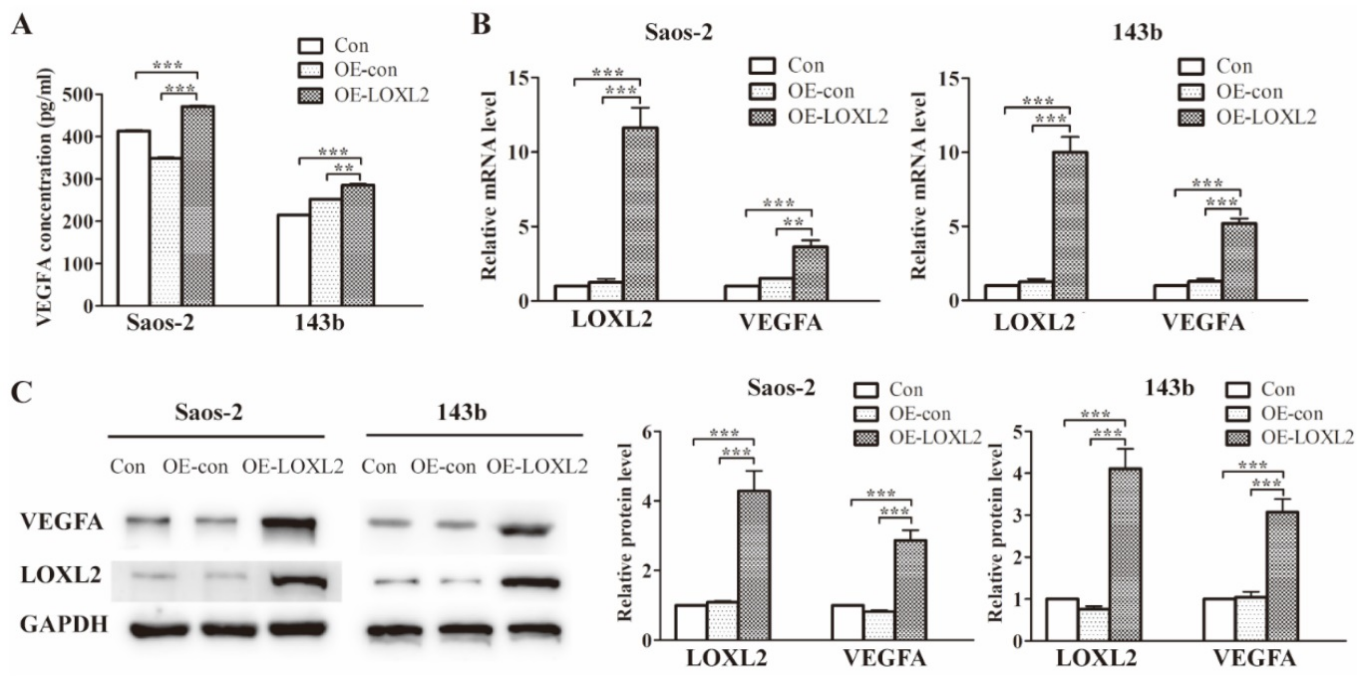
Treatment with DHA reduces LOXL2 expression and the LOXL2/VEGFA pathway.(A) Concentrations of VEGFA secreted into the medium collected from Saos-2 and 143b cells infected with overexpression vector were detected by ELISA. (B) Overexpression efficiency of LOXL2 and effect of altered LOXL2 expression on VEGFA mRNA levels in Saos-2 and 143b cells, as determined by qRT-PCR assay. (C) Overexpression efficiency of LOXL2 and effect of altered LOXL2 expression on VEGFA protein levels in Saos-2 and 143b cells, as determined by western blot assay. (** *P* < 0.01, *** P < 0.001 compared with the control).

Previous research had identified the association between LOXL2 and VEGFA expression. To verify this association in OS cells, we enforced LOXL2 overexpression in Saos-2 and 143b cells. The VEGFA secretion significantly increased in the LOXL2 overexpression group compared with that in the negative control group (Figure 3A). Similarly, the qRT-PCR and western blot assays revealed the VEGFA expression could be regulated by LOXL2 at the mRNA and protein levels (Figure 3B and Figure 3C).

### DHA widely interferes with the lipid metabolism in OS cells

**Figure 4:**
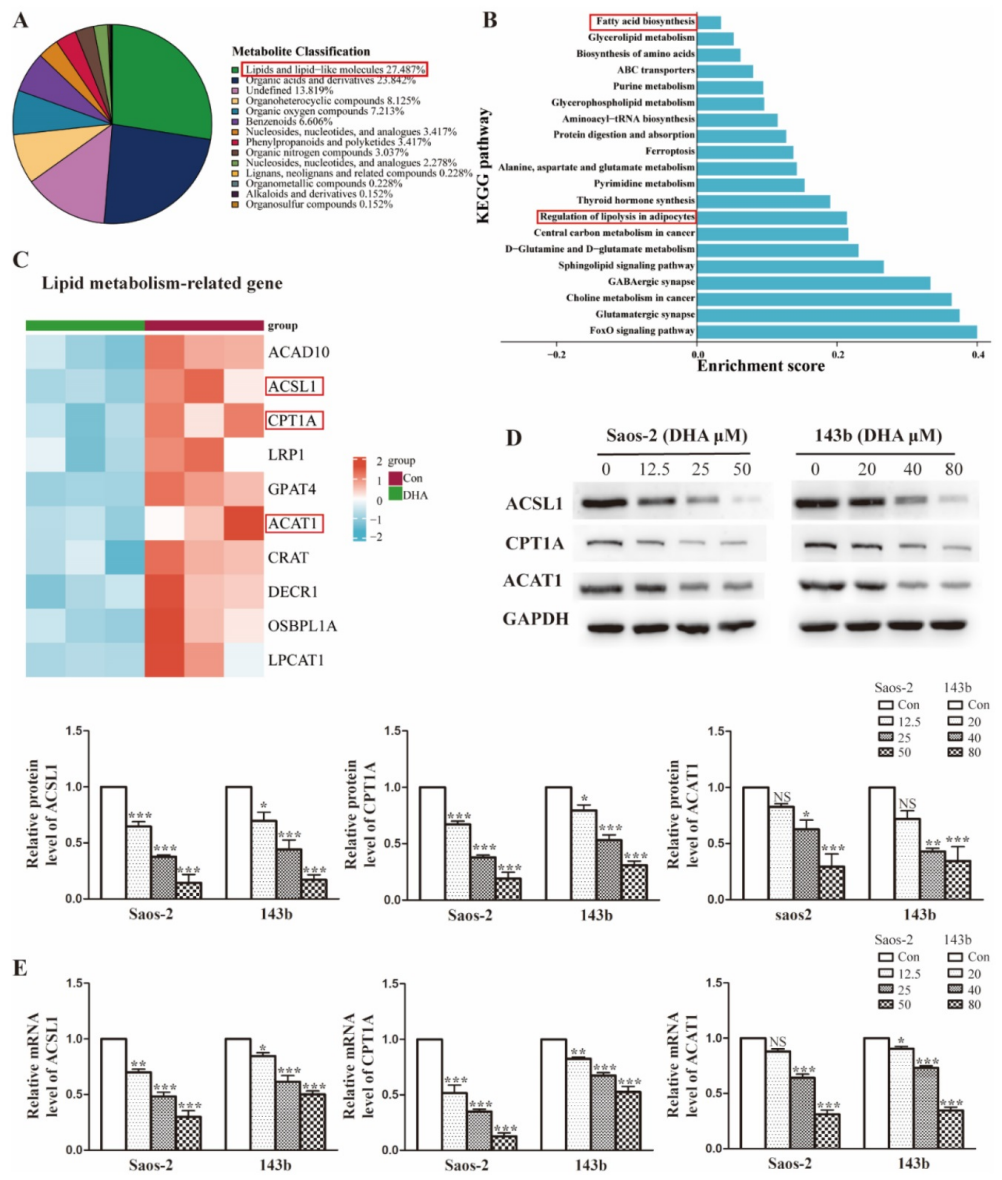
DHA widely interferes with lipid metabolism in OS cells. (A) Superclass pie-chart showed that 27.4% of differentially expressed metabolites are involved in the lipid metabolic process. (B) KEGG enrichment analysis for metabolism data, showing the top20 significant altered metabolic pathways after DHA treatment. (C) Heatmap of the top10 lipid-associated proteins after DHA treatment, proteins marked in red are involved in the FAO pathway. (D-E) The expression of FAO-related protein ACSL1, CPT1A, and ACAT1 was determined by qRT-PCR and western blot assay.

The enrichment analysis of DEPs revealed that DHA treatment leads to widespread changes in lipid metabolic programs in OS cells. To identify the specific lipid programs affected, an untargeted metabolomic was performed. As shown in Figure 4A, approximately 27.5% of affected metabolites were lipid-related molecules, suggesting that the lipid metabolism may be widely influenced after DHA treatment. To explore the specific pathways in detail, KEGG function analysis was performed. We showed that the top 20 KEGG pathways and lipid metabolism-associated pathways included fatty acid biosynthesis and regulation of lipolysis (Figure 4B). In the proteomics results, we found that the lipid metabolism-related proteins were markedly downregulated after DHA treatment and showed the most downregulated proteins in the heatmap (Figure 4C). Notably, the common proteins, such as ACSL1(acyl-CoA synthetase long-chain family member 1), CPT1A (carnitine palmitoyltransferase 1A), and ACAT1 (acetyl-Coenzyme A acetyltransferase 1), were those associated with the processes of fatty acid metabolism. As confirmatory tests, we performed qRT-PCR and western blot assays to assess the expression change of ACSL1, CPT1A, and ACAT1 in OS cells treated with DHA (Figure 4D and Figure 4E). The results were consistent with the -omic analysis and confirmed that DHA can affect lipid metabolism, especially the FAO (fatty acid oxidation) process in OS cells.

### DHA synergizes with VEGFR-TKIs to inhibit OS cell proliferation

**Figure 5:**
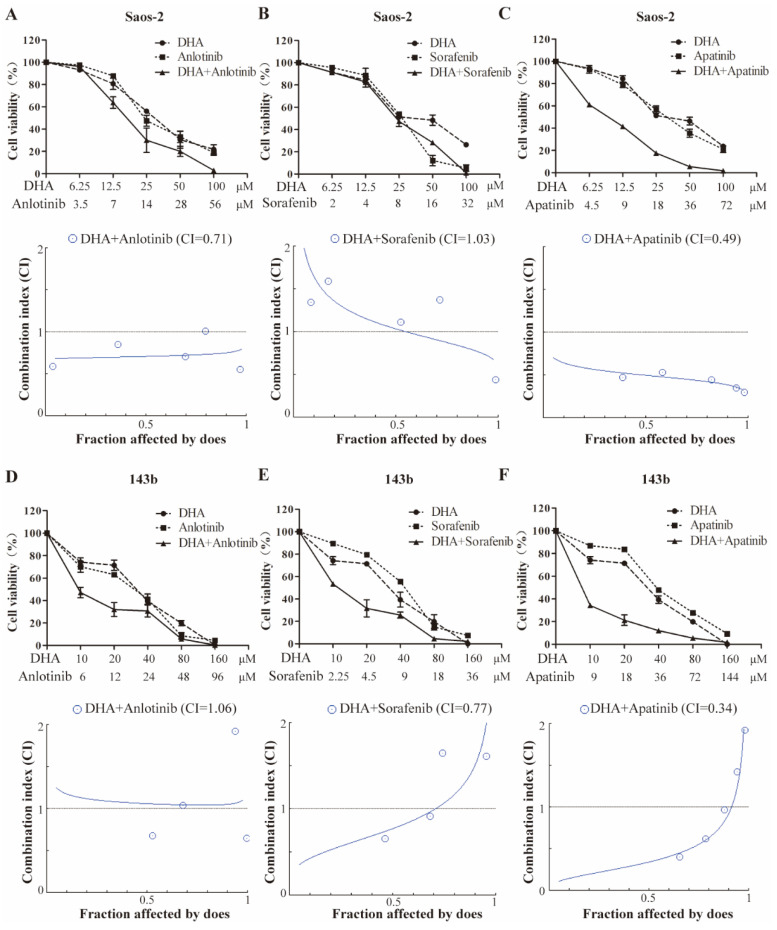
DHA synergizes with VEGFR-TKIs to inhibit OS cell proliferation. Growth inhibition is induced by the VEGFR inhibitors and DHA in combination. Saos-2 (A-C) and 143b (D-F) cells were treated with the indicated doses of anlotinib, sorafenib, and apatinib in combination with DHA for 24 hours, and the cell viability assay was subsequently performed. Data are presented as a percentage of the control. Median effect analysis (CalcuSyn software) was used to evaluate the interaction between the drug combinations.

The half-inhibitory concentrations (IC_50_) of DHA, apatinib, anlotinib, and sorafenib on the growth of OS cells were determined after 24-hour exposure (Table 1). These IC_50_ values were used to assess the potential synergy by the Chou-Talalay equipotent fixed ratio method (Figure 5). Combined treatment of Saos-2 cell with DHA and either anlotinib or apatinib resulted in markedly enhanced inhibition compared with DHA alone (Figure 5A-5C). In 143b cell, DHA similarly had a synergetic effect when combined with either sorafenib or apatinib (Figure 5D-5F).

### DHA enhances cell growth-inhibitory effects of apatinib in vitro and in vivo

**Figure 6:**
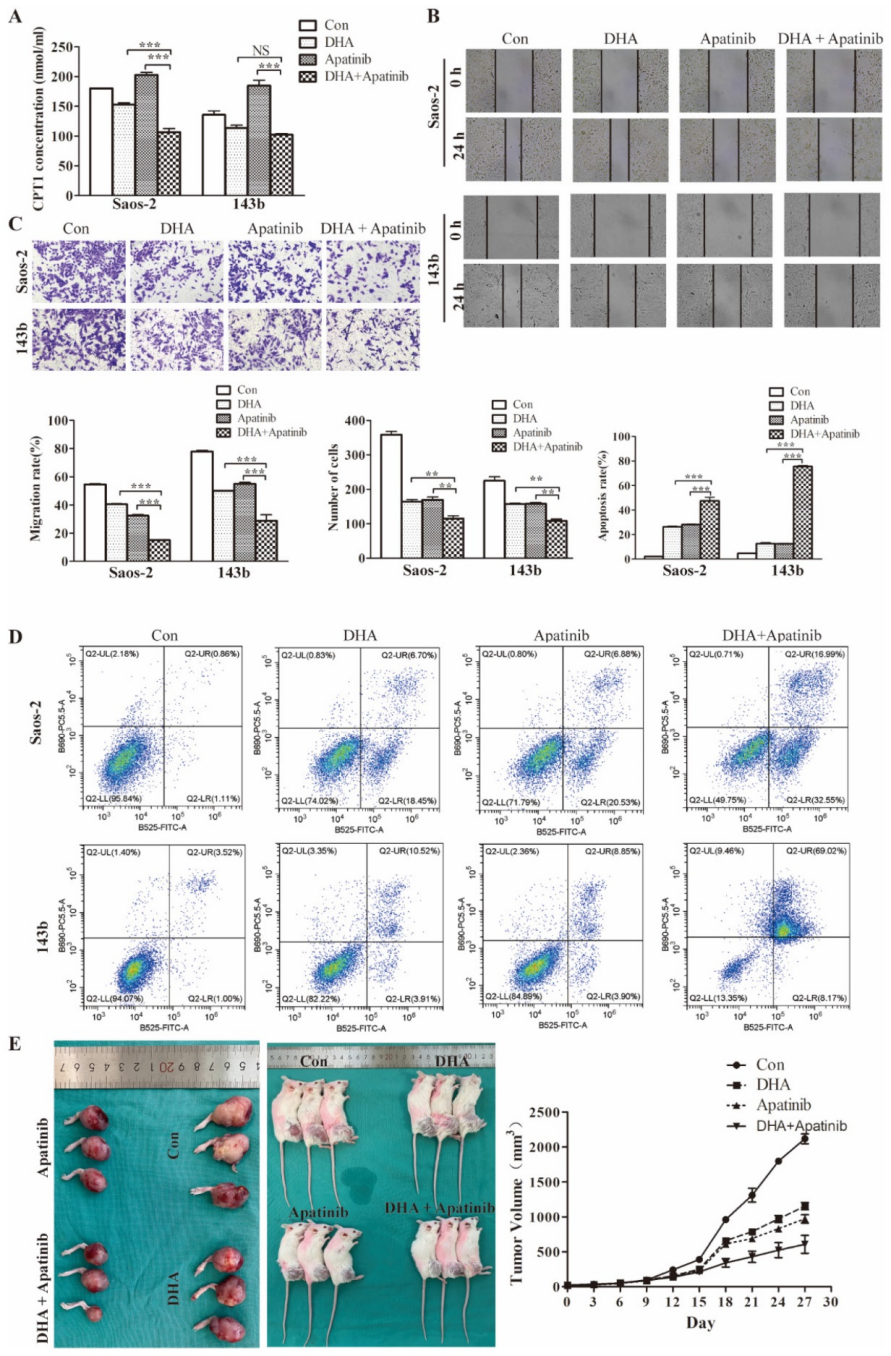
DHA enhances cell growth inhibition by Apatinib in vitro and in vivo. The DHA and apatinib were combined at a ratio of 1:1 in both Saos-2 and 143b cells. Saos-2 cell were treated with DHA (25.41 μmol/L) and apatinib (18.03 μmol/L) for 24 h. 143b cell were treated with DHA (41.98 μmol/L) and apatinib (36.11 μmol/L) for 24h. (A) Concentrations of CPT-1 collected from Saos-2 and 143b cells treated with DHA and apatinib alone or in combination were detected by ELISA. DHA combined with apatinib inhibited the CPT-1 activity compared to apatinib alone. (B) The wound healing assay showed that the combination impaired wound healing more than DHA or apatinib alone. (C) The transwell assay indicated that the combination has a stronger effect on invasion compared to DHA or apatinib alone. (D) OS cells were stained with Annexin V-FITC and PI and then subjected to flow cytometric analysis of cell apoptosis; the combination induced more apoptosis. (E) 143b tumor-bearing mice were treated intraperitoneally with DMSO, apatinib or DHA alone (50 mg/kg), and DHA (25 mg/kg) combined with apatinib (25 mg/kg). The tumor volumes were measured every 3 days, and growth curves of the tumors were drawn. (apatinib group vs control * *P* < 0.05, DHA group vs control group * *P* < 0.05, apatinib + DHA group vs control group *** *P* < 0.001).

Commonly, anti-VEGF therapy induces tumor tissue hypoxia, consistent with antiangiogenic drugs decreasing vascularity and blood perfusion. Moreover, it has been demonstrated that anti-VEGF treatment triggers reprogramming of lipid metabolism in the tumor microenvironment, which confers resistance to antiangiogenic drugs. Consequently, it was plausible that lipid metabolic changes induced by DHA might potentiate antiangiogenic drugs. As the above data showed, DHA inhibits the fatty acid oxidation process in OS cells. We used the CPT-1 ELISA kit to test the effect of DHA, apatinib, and the combination on fatty acid oxidation activity. As shown, apatinib on its own could activate the fatty acid oxidation process, whereas in combination with DHA this activation was reversed (Figure 6A).

Moreover, wound healing, transwell, and flow cytometry assays were performed to further examine the synergistic effect of DHA combined with apatinib in relation to migratory, invasive, and apoptotic activities (Figure 6B-6D). The results suggested that the combination treatment group had greater effects on migration, invasion, and apoptosis than single-agent treatment. Then we explored the antitumor activity of this combination in 143b-derived murine xenografts (Figure 6E). As expected, the combined treatment resulted greater delay in tumor growth compared with the group treated with apatinib alone.

## Discussion

Despite combining surgery and chemotherapy as the standard of treatment, the overall survival rate of OS patients with recurrence or metastasis is still poor, partly owing to heterogeneous histological and drug resistance. Consequently, there is an urgent need to identify novel therapies, especially drug combinations that exert effects through multiple mechanisms, in order to enhance the efficacy of the current treatment strategies. DHA is derived from artemisinin, has low toxicity, and is in worldwide use as an anti-malarial drug. Previous studies showed that DHA can inhibit the growth of various tumor cells and induce apoptosis via multiple signaling pathways 11,12,13,14 and effects on osteosarcoma were recently reported by Shen et, al 8. In this study, we similarly treated OS cells with DHA and verified that it can strongly inhibit the growth, migration, and invasion of OS cells in vitro and in vivo, thereby verifying potential for application in novel therapies.

To further explore the underlying mechanism, we used proteomics and metabolomics to analyze the fundamental changes in DHA-treated OS cells. Multi-omics had not previously been used to explore the key regulators of DHA effects in OS and the proteomic results showed that LOXL2 expression level was downregulated nearly threefold, which captured our attention. Lysyl oxidase-like 2 is a member of a family of lysyl oxidase enzymes that contribute to collagen and elastin crosslinking to ensure the structural integrity and function of several tissues 15. LOXL2 level has been reported to correlate with poor prognosis of several solid cancers, such as breast cancer 16, colon cancer 17, and gastric cancer 18. LOXL2 participates in the repression of E-cadherin CDH1, a hallmark of the epithelial to mesenchymal transition (EMT) that is believed to amplify tumor aggressiveness, suggesting that it may play a role in tumor progression 19. Kazuhiko et, al. suggested that LOXL2 is probably a tumor promoter in OS that favors bone formation and promotes a more aggressive osteoblastic phenotype; cFos/LOXL2 co-expression correlated with decreased survival of OS patients 20. Given the above research basis, it was plausible to assume that DHA may exert inhibitory effects in OS cells by limiting LOXL2 expression. In this study, we used qRT-PCR and western blot assays to show that DHA did inhibit LOXL2 expression in OS cells and the inhibition was dose-dependent. Moreover, several studies have indicated that LOXL2 may act as an adaptive response protein to promote VEGFA secretion and tumor angiogenesis. A few studies suggested that LOXL2 may directly initiate angiogenesis on collagen IV scaffolding 21. Additionally, by using in vitro and in vivo angiogenesis assays, Shelly et al. further confirmed that LOXL2 plays a direct role in angiogenesis and indicated an effect on VEGF signal transduction and perhaps additional angiogenic factors 22. Recently, Peng et al. pointed out that LOXL2 can interact with GATA to enhance VEGFA expression and promote angiogenesis and tumor growth in cholangiocarcinoma 23. Here, in order to verify that LOXL2 could affect VEGFA expression in OS we overexpressed LOXL2 in Saos-2 and 143b cells, and as expected both qRT-PCR and western blot results showed that overexpressing LOXL2 upregulates the mRNA and protein levels of VEGFA. ELISA assays confirmed the positive correlation between LOXL2 and VEGFA secretion in OS cells.

Our metabolomics data showed that DHA induced substantial changes in the lipid metabolism of OS cells. Metabolic reprogramming of tumor cells can be considered to be a hallmark of cancer 24; metabolic changes in adipose tissue and free fatty acid (FFA) significantly contribute to tumor cell survival, proliferation, and migration 25. Unlike most healthy cells, proliferation of tumor cells is uncontrolled, hence, they must continuously produce the necessary energy 26. Besides relying on the Warburg effect and glutamine consumption, tumor cells can also activate endogenous lipid biosynthesis by taking in exogenous lipids 27. It is plausible that DHA might also exert a partial anti-tumor effect by interfering with the lipid metabolism of OS cells. To explore this possibility, we re-analyzed the proteomic data. As expected, proteins related to lipid metabolism were significantly changed in OS cells treated with DHA. qRT-PCR and western blot assays demonstrated that there was substantial inhibition of proteins such as ACSL1, CPT1a, and ACAT which are key functional enzymes in the fatty acid oxidation process. Assays of fatty acid oxidation activity confirmed inhibition by DHA, which would not only directly affect OS cell growth but also influence the cell's response to antiangiogenic drugs.

Although DHA and other artemisinin derivatives are known to induce apoptosis in tumor cells, they have rarely been applied to the clinical treatment of tumors. Duan et, al. reported a strong synergy between DHA and oxaliplatin in generating reactive oxygen species (ROS) and anticancer activity 28. DHA potentiation of the antitumor effect of gemcitabine in pancreatic cancer and hepatoma cells has also been reported 29,30. Given our above results showing that DHA regulates LOXL2/VEGFA expression and affects the fatty acid oxidation program, we presume that DHA may exert a synergistic anti-tumor effect with antiangiogenic drugs.

Small molecular inhibitors that target drivers of angiogenesis are becoming standard therapies for some cancers. Although antiangiogenic drugs are conventionally used in patients with tumors, they target multiple angiogenic signaling pathways and may not necessarily result in greater improvements in long-term survival compared with monospecific therapies 31. In addition, patients treated with VEGFR-TKIs often suffer from intrinsic drug resistance that hinders the therapeutic efficacy. We know that antiangiogenic drug treatment can decrease tumor vascular density to a low level, thereby limit the nutrients required for tumor growth to achieve the anti-tumor effect. However, reduction of blood vessels is prone to creating a higher hypoxic environment in tumor tissue 32. And tumor hypoxia can intensify the secretion of growth factors and cytokines which reduce the drug targets and may facilitate the development of resistance. A hypoxic tumor microenvironment can also change the invasive ability and drug response of tumor cells 33. Bensaad et. al. have shown that anti-VEGF treatment can induce lipid transport and storage in tumor cells via an HIF-1-dependent hypoxia mechanism 34. Anti-VEGF treatment also triggers oxygen and nutrient exhaustion through inhibition of angiogenesis and converts a glucose-dependent metabolism to a lipid-dependent metabolism, which ensures tumor growth even in the presence of few vessels 35 and cancer lipid metabolism has been proposed to confer antiangiogenic drug resistance. Our study clearly showed that DHA inhibits the lipid metabolic pathway in OS cells. Combined with our finding that DHA affects VEGFA expression by directly regulating the LOXL2, it was reasonable to presume a synergistic anti-tumor effect of DHA combined with VEGFR-TKIs. We treated Saos-2 and 143b cells with DHA combined with apatinib, alnotinib, and sorafenib respectively following by Chou-Talalay method. The combination index (CI) results suggested that treatment with DHA had a synergistic effect to a different degree when combed with different angiogenic drugs since DHA combined with apatinib had the strongest synergy in both cell types that were tested. Assays in vitro and in vivo demonstrated DHA enhanced the efficacy of apatinib in inhibiting OS cells migration, invasion, and apoptosis.

To summarize, our study shows that DHA significantly inhibits OS cells proliferation, migration, and invasion by suppressing the expression of LOXL2, a tumor-promoting factor, and reducing VEGFA expression by directly regulating LOXL2. In addition, we showed that DHA impaired the fatty acid oxidation program in OS cells, thereby delaying the development of resistance to antiangiogenic drugs. These findings support the view that DHA potentiates the anti-tumor activity of VEGFR-TKIs in OS. DHA may be worthy of exploration in combination therapy with antiangiogenic drugs. However, the explicit molecular mechanisms that underlie antiangiogenic drug resistance are not yet clear enough; for example, the lipid metabolism-dependent mechanism is responsible in the animal model, but its relevance is uncertain for cancer patients and anti-lipolysis agents have not been tested in clinical treatment of cancer patients. As a first step, tissue samples resected from OS patients who have developed antiangiogenic drug resistance might be used to explore the potential for synergy demonstrated here.

## Figures and Tables

**Table 1:** CI and IC_50_ values at 24 h for DHA and TKI combinations

Cell line	DHA (IC_50_) μmol/L	VEGFR-TKI (IC_50_) μmol/L		CI
143b	41.98	apatinib	36.11	0.34
		anlotinib	24.58	1.06
		sorafenib	9.05	0.77
Saos-2	25.41	apatinib	18.03	0.49
		anlotinib	14.63	0.71
		sorafenib	7.99	1.03

## Abbreviations

OS: osteosarcoma
LOXL2: collagen-modifying enzyme lysyl oxidase-like 2
DHA: dihydroartemisinin
VEGFA: vascular endothelial growth factor A
VEGF: vascular endothelial growth factor
VEGFR-TKIs: vascular endothelial growth factor receptor-tyrosine kinase inhibitors
ACSL1: miRNA response element
ABC: acyl-CoA synthetase long-chain family member 1
CPT1A: carnitine palmitoyltransferase 1A
ACAT1: acetyl-Coenzyme A acetyltransferase 1
GO: Gene Ontology
DEPs: differentially expressed proteins
KEGG: Kyoto Encyclopaedia of Genes and Genomes
VIP: variable importance in the projection
GATA: globin transcription factor
FFA: free fatty acid
ROS: reactive oxygen species
CI: combination index
